# European Joint Clinical Assessment PICO Scoping Process: Analysis of Current Approaches and Recommendations

**DOI:** 10.3390/jmahp14010003

**Published:** 2025-12-29

**Authors:** Kalpana D’Oca, Eline Darquennes, Chloé Garrigues, Aristeidis Draganigos, Natalie Steck

**Affiliations:** 1 MSD (UK) Limited, London EC2M 6UR, UK; 2 MSD Belgium, 1170 Brussels, Belgium; 3 MSD France, 92800 Puteaux, France; chloe.garrigues@msd.com; 4 MSD Greece, 17456 Alimos, Greece; aristeidis.draganigos@msd.com; 5 MSD Innovation & Development GmbH, CH-8058 Zurich, Switzerland; natalie.steck@msd.com

**Keywords:** EU HTA, JCA, PICO scoping, JCA PICO consolidation

## Abstract

The PICO framework determines the scope of the Joint Clinical Assessment (JCA) under the EU HTA Regulation (EU HTAR), with PICO consolidation being a critical final step of the scoping process. Due to limited clarity on how consolidation works in practice, Health Technology Developers (HTDs) may simulate PICO scoping as a strategic tool to guide the development of robust JCA submissions. A review of 14 publications, representing 35 individual PICO exercises across 20 indications (74% in oncology), showed an average of 7 countries participating per exercise and 8 consolidated PICOs per analysis. A separate PICO scoping simulation focused on a first-line immuno-oncology treatment for metastatic non-small cell lung cancer (mNSCLC) generated 67 PICOs, reflecting the anticipated perspectives of 25 countries, largely driven by biomarker and histology-based sub-populations. The limited number of published examples and country participation restricts the ability to draw clear conclusions or confidently predict the output of PICO scoping in a real life JCA processes. The simulation also raised questions about whether all sub-populations should be included or consolidated further. Overall, there is a need for greater clarity in the JCA PICO scoping process, in particular the consolidation step, to facilitate high-quality evidence generation and support the EU HTAR to meet its goals of efficiency, transparency, and equity in health technology evaluation across Europe, along with more consistent patient access.

## 1. Introduction

The European Union’s Regulation on Health Technology Assessment (EU HTAR) [1] was adopted in December 2021 and came into force on 11 January 2022. A three-year transition period followed, leading to an application date of 12 January 2025 with new oncology treatments and advanced therapy medicinal products (ATMPs) in scope immediately. Orphan medicinal products and all new medicinal products will become eligible in 2028 and 2030, respectively [2].

The nature of Health Technology Assessment (HTA) in the European Union (EU) is undergoing a transformative shift with the introduction of Joint Clinical Assessments (JCAs), as mandated by the EU HTAR [1]. JCAs represent a key pillar of the EU HTAR, encompassing a pan-EU clinical assessment that provides a scientific analysis of clinical evidence concerning the relative effects of a health technology (a medicinal product or a medical device) on health outcomes. The scope of the JCA, as determined by the PICO (population, intervention, comparator, and outcome) framework, will focus on clinical domains only, without any value judgements or conclusions on reimbursement. Due consideration [1,2] should be given to the output of the JCA process at a national level during the country-specific assessments and appraisals that follow; consequently, analyses included in the JCA can inform decisions pertaining to budget and resource allocation in healthcare, including pricing or reimbursement of health technologies.

The JCA aims to streamline the clinical evaluation process for new health technologies across member states, reducing redundancies and enhancing the efficiency and consistency of HTA outcomes. Through its implementation, the JCA is focused on four key objectives [3] including harmonisation of HTA processes, improved access to innovations, resource efficiency, and transparency and trust, with the involvement of seven groups of stakeholders, namely the Member State Coordination Group on Health Technology Assessment (HTACG), the JCA Subgroup, Health Technology Developers (HTDs), Individual Experts (patients, clinical experts and other relevant experts), the HTA Secretariat in the European Commission and the European Medicines Agency (EMA).

The increase in collaboration, driven by fostering the sharing of data and methodologies relating to HTA among member states, strives to reduce the duplication of effort, optimise the use of resources and reduce the burden on HTDs [4]. By consolidating clinical evidence across member states and standardising the approach for assessment of clinical and safety data, JCAs aim to create a more consistent, harmonised approach to HTA methodology across EU countries. This should avoid evaluations occurring across member states based on inconsistent criteria, and instead reduce discrepancies in HTA outcomes, which can otherwise lead to inequitable access to health technologies across the EU [5]. The sharing of expertise and knowledge across member states can lead to more robust and comprehensive evaluations, and the adoption of a standardised approach to JCA, which is transparent and evidence-based, can help build trust in the new process among all key stakeholders involved. The precise impact on national HTA submission process is yet to be seen, but with the introduction of JCA, the ambition is that it will contribute to faster patient access to new and innovative therapies [3].

**Figure 1:**
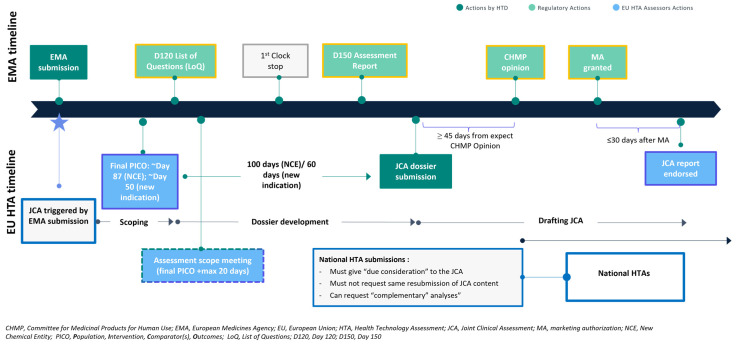
Timelines for JCA process relative to EMA process milestones.

The JCA involves a structured process with defined milestones and timelines, which are anchored to key milestones in the EMA’s regulatory process (Figure 1). It begins with the PICO scoping phase, using the PICO framework, which incorporates input received from all EU member states to arrive at a consolidated set of PICOs that form the assessment scope and defines the parameters of the assessment. Following PICO scoping, HTDs have only a 100 day period (or 60 days for Type II variations) to finalise their JCA dossier prior to submission to the HTA CG. The final step involves the coordination of the assessment by the assessor and co-assessor, resulting in the publication of a JCA report [6].

The final JCA report should be endorsed within 30 days of the marketing authorisation being issued, providing a comprehensive assessment of the clinical and safety data relating the health technology under consideration. The final report is shared with all EU member states and made publicly available, ensuring that the findings are accessible for national decision-making processes.

**Figure 2:**
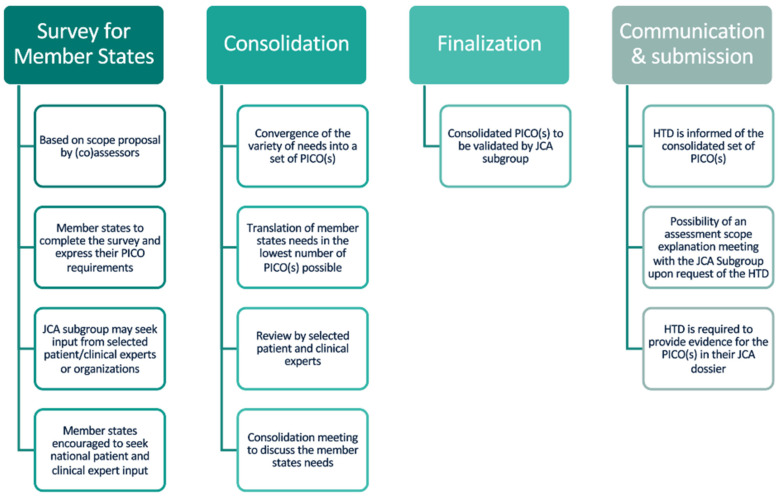
Key PICO scoping process steps in JCA.

JCA PICO scoping (including consolidation) is central to the JCA process, as it defines the scope and parameters of the clinical evaluation, thereby ensures that the clinical questions addressed are relevant. Guidance on the scoping process confirms that to achieve the lowest possible number of PICO(s) during the consolidation phase, the assessor and co-assessor might contact the MS member of the JCA subgroup to “*clarify open questions resulting from the PICO survey and discuss options for consolidation, especially if a specific PICO or a PICO component is only requested by one MS*” [7] (p. 19). The key steps in the official PICO scoping process are summarised below in Figure 2.

From the assessor and co-assessor perspective, a clear set of PICO(s) provides structure to the scope of a JCA and facilitates their focused evaluation of the evidence based on a clear, well-defined research question [8]. Similarly, from the HTD perspective, a well-defined set of PICO(s) aids the dossier preparation process and facilitates the submission of targeted evidence that directly addresses the key comparisons of interest determined by assessors/co-assessors, thereby minimising the likelihood of receiving requests for additional evidence during the JCA process or alternatively during subsequent national HTAs. Given the importance of PICO scoping within the JCA process, HTDs are motivated to use simulation of this process as a strategic tool to guide the development of high quality, targeted and robust JCA dossiers.

With this publication, we aimed to gather insights from multi-country PICO scoping process examples, by (1) reviewing previously published exercises and (2) conducting a PICO scoping simulation designed to follow the official JCA guidance from the HTA CG. These insights help form recommendations which aim to further clarify the process for involved stakeholders during future JCA PICO scoping exercises, with the intent of supporting the objectives of the EU HTAR to result in expedited patient access.

## 2. Methods

Previous multi-country PICO scoping exercises were identified based on (1) publications that provide examples, including published HTA reports, where member states’ PICOs were extracted and consolidated based on EUnetHTA guidelines, or (2) published exercises conducted by EUnetHTA, EFPIA or the EU HTA CG. Publications were found through keyword searches conducted in 2024, without any exclusion criteria applied based on publication date. The scope of the keyword searches was intentionally limited to ensure that the examples included were based on authoritative, widely recognised sources that reflect structured and transparent methodologies aligned with European HTA processes.

Identified publications were reviewed to establish the number of individual PICO exercises conducted and which indications they concerned. The average number of countries taken into consideration in each exercise was calculated. The number of consolidated PICOs per analysis was extracted, and the average number of consolidated PICOs across all identified exercises in a given indication was calculated.

Separately, we ran a JCA PICO scoping simulation during the second half of 2024, which was based on the official JCA scoping methodology described in the guidance published in November 2024 [7], but without soliciting direct input from clinicians or patients. The exercise focused on an upcoming asset planned for the first-line treatment of metastatic non-small cell lung cancer (mNSCLC) in adults without EGFR, ALK or ROS genomic tumour aberrations, not restricted by histology or PD-L1 status. This indication was selected due to it being a dynamic therapy area with a busy treatment landscape, also characterised by sub-population considerations concerning potential different biomarkers and histologies of relevance.

Local affiliates providing perspectives covering 25 countries were invited to participate in the exercise to reflect the different perspectives across the region. The exercise was conducted in a stepwise approach, as defined in the guidance [7]: firstly, to replicate the initial task of the assessor/co-assessor in the JCA PICO scoping process, a preliminary set of PICOs was drafted based on background research informed by published clinical guidelines (e.g., ESMO [9,10]). Subsequently, a survey was developed and launched to share the preliminary PICOs with local affiliates, with a request that they review and provide feedback based on the anticipated perspectives of HTA bodies in 25 European countries. Additional PICOs could also be proposed by the local affiliates. Feedback was requested in a predefined timeframe, following which a meeting was organised to attempt to consolidate the outcomes of the survey and arrive at a defined set of anticipated PICOs.

## 3. Results

### 3.1. Published Multi-Country PICO Scoping Exercises

14 [11,12,13,14,15,16,17,18,19,20,21,22,23,24] publications were identified through keyword searches and included for review, as summarised in Table 1. Out of the 14 identified publications:

- Seven exercises [11,12,13,14,15,20,21] relied mainly on published HTA reports from where each member state’s proposed PICOs were extracted. The resulting PICOs were then consolidated as per EUnetHTA guidelines
- Three exercises [16,17,18] were conducted by EUnetHTA for medicinal products that had already obtained a positive CHMP opinion
- One exercise [19] was conducted by EFPIA in collaboration with Evidera, where both published HTA documents as well as local clinical guidelines were used in the identification of local PICOs for three products/indications
- Three exercises [22,23,24] were conducted by the JCA subgroup of the HTA CG

**Figure 3:**
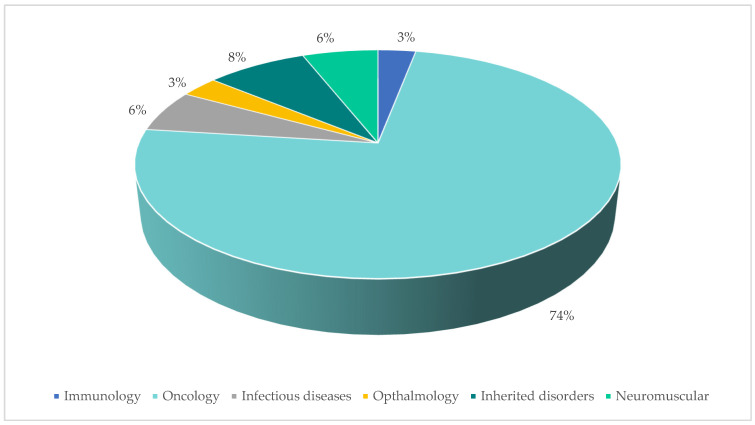
PICO exercises by Therapy Area.

**Figure 4:**
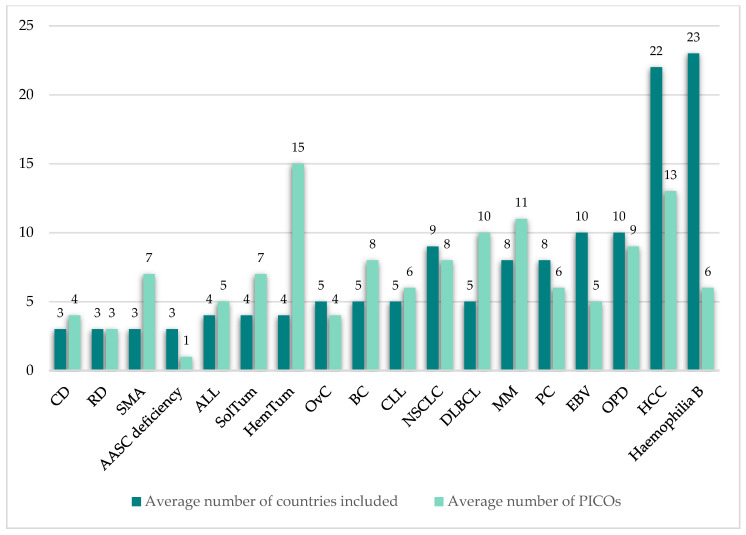
Per indication, average number of countries included and average number of PICOs identified.

**Figure 5:**
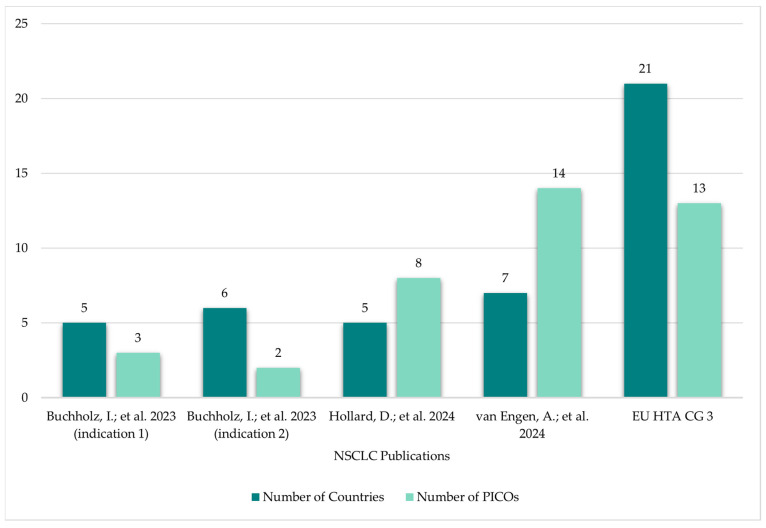
Number of countries involved and PICOs identified: NSCLC PICO scoping examples [12,13,14,23].

The 14 included publications described in total 35 individual PICO exercises conducted in 21 indications. The vast majority of identified PICO exercises (74%) were in the oncology therapy area (Figure 3). The average number of countries taken into consideration across the identified exercises was 7 (range 3–23; Figure 4) and the average number of consolidated PICOs per analysis conducted was 8 (range 1–15; Figure 4). In therapy areas such as NSCLC where multiple PICO exercises were identified from different sources, the number of countries involved in each exercise and the resulting number of PICOs was variable (Figure 5).

### 3.2. Results from JCA PICO Scoping Simulation in NSCLC

**Figure 6:**
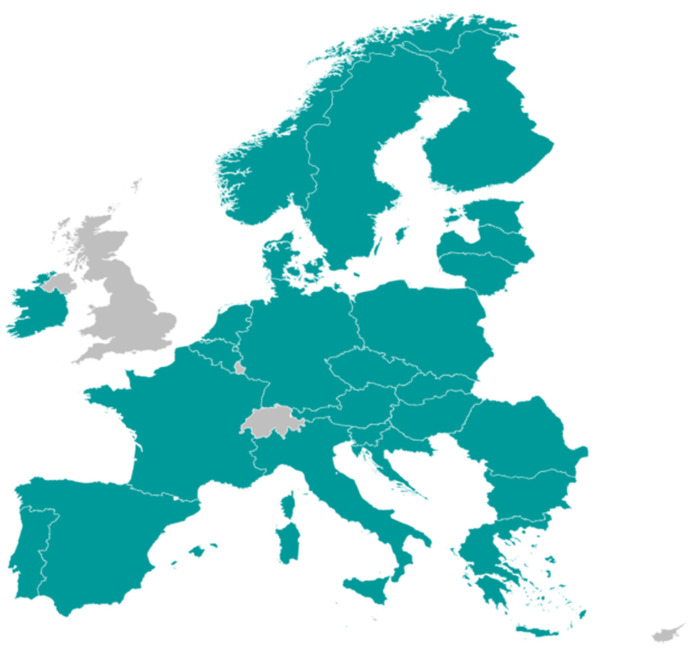
Country perspectives provided in the JCA PICO scoping simulation.

Local affiliates providing perspectives covering 25 countries participated and responded to the JCA PICO scoping performed during the second half of 2024 (Figure 6).

Following a review of the responses, a total of 67 anticipated PICOs were defined for the NSCLC indication, based on the understanding that one PICO comprises one population, one intervention (or combination), one comparator (which can include more than one treatment), and at least one outcome. Table 2 below provides an overview of the number of PICOs in the full population versus sub-populations. The high number of sub-populations was driven based on histology (squamous and non-squamous) combined with a variety of PD-L1 biomarker cut-off values.

**Figure 7:**
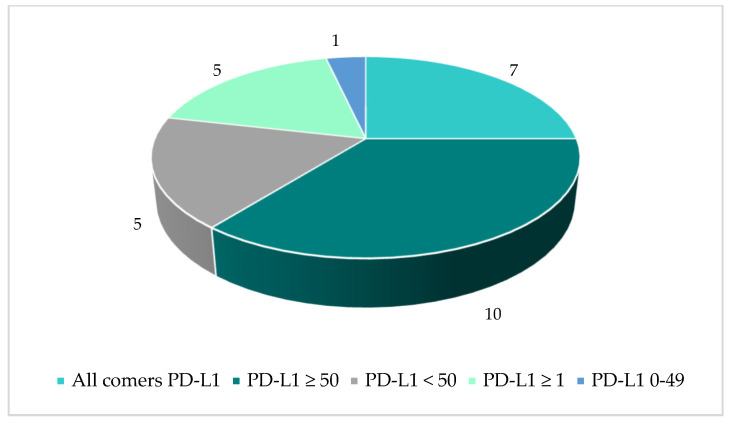
All histologies: Number of PICOs per population.

**Figure 8:**
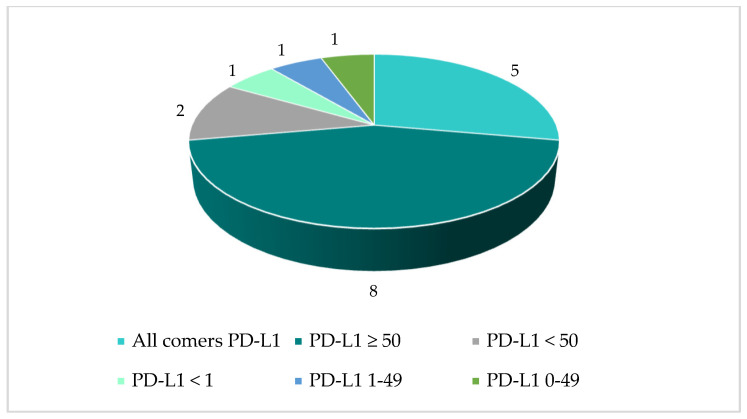
Squamous histology: Number of PICOs per population.

**Figure 9:**
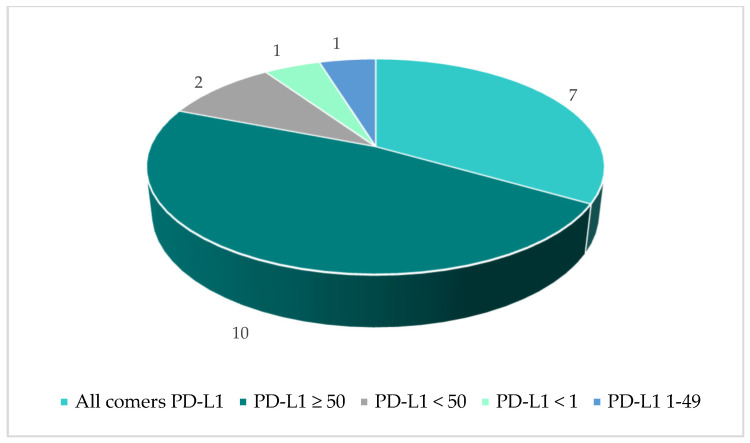
Non-squamous histology: Number of PICOs per population.

Some responses to the PICO scoping simulation survey included requests for additional comparators and/or additional biomarker cut-off values (leading to new sub-populations) from what had been pre-defined, due to local clinical practice and specific member states’ needs. The number of identified PICOs per population and histology are provided in Figure 7, Figure 8 and Figure 9. It may be questioned whether these PICOs would be retained by the assessor and co-assessor during the official EU process.

## 4. Discussion

The JCA process aims to ensure that clinical evaluations are methodologically robust and aligned with stakeholder needs, which is critical in the context of the European Union’s evolving landscape, where harmonisation and transparency are key objectives under the EU HTAR [1]. The PICO scoping process, including the consolidation step, provides a structured approach for defining the clinical questions that guide the assessment of health technologies. This involves collaboration among member states, HTA bodies, and relevant stakeholders to identify and agree upon the most relevant clinical parameters for evaluation. Under the former EUnetHTA framework, participation in joint work, such as scoping exercises for assessments, was entirely voluntary. National HTA bodies could choose whether to engage in these activities, and there was no legal obligation for manufacturers or agencies to follow the outputs. This voluntary approach often led to variability in participation and limited predictability for industry planning. In contrast, the EU HTAR introduces a mandatory system for JCA. The scoping process under the EU HTAR is a formal, regulated step within JCA procedures, governed by procedural and methodological guidelines adopted by the HTA Coordination Group and the resulting scope is binding for the joint assessment. This shift ensures consistency, transparency, and legal enforceability across Member States, unlike the previous voluntary EUnetHTA model.

The JCA Implementing Act [25] clearly articulates the vision of member states’ input into the PICO scoping process, which should be to translate their clinical requirements into the fewest possible PICO sets. Nevertheless, depending on the type of therapy, the condition being treated and the country-specific treatment pathway, the number of potentially relevant PICOs identified by the concerned member states during the scoping process could be highly variable, taking into consideration the definition that one PICO comprises one population, one intervention (or combination), one comparator (which can include more than one treatment), and at least one outcome. While published guidance [7] outlines a consolidation process to refine these into a minimal set, practical challenges remain. Findings from the PICO scoping exercises described in the results section of this paper highlight limited experience and transparency in the process, making it difficult to reliably predict outcomes in real-world JCA settings.

Although consensus guidelines for a treatment of a particular condition may help guide the drafting of the initial set of proposed PICOs, healthcare practices can differ significantly across EU member states due to variations in clinical guidelines, treatment pathways, standard of care, and national health priorities, making it challenging for (co-)assessors to define PICOs that are both scientifically rigorous and broadly applicable across jurisdictions [26]. This diversity can lead to multiple PICOs being proposed, especially if off-label comparators are considered, increasing complexity for HTDs in generating suitable evidence to address the assessment scope [19]. Additionally, the rapid pace of innovation can often outstrip the availability of robust comparator data, adding to the challenge of preparing appropriate comparative evidence.

The additive PICO approach, as outlined previously by EUnetHTA 21 [13], could lead to a significant number of PICOs if applied in the future exercises. While this method aims to capture diverse national perspectives, a high number of PICOs included in the assessment scope at EU level increases the complexity for dossier preparation and assessment. To maintain a focused and efficient JCA process and to avoid less-essential PICOs being included in the assessment scope, it is essential that member states only propose PICOs that are truly relevant from a national perspective, when responding to the PICO scoping survey.

Examples from published multi-country PICO scoping exercises demonstrate variability in the number of PICOs identified per exercise, influenced by the therapy area and number of HTA bodies involved. Findings from analyses based mainly on published HTA reports may underestimate the true number of consolidated PICOs, as they involve only a limited number of EU member states. Since the number of PICOs identified may be impacted by broader country participation (Figure 6), relying on a small or representative sample may be inadequate to confidently predict the full set of PICOs likely to emerge during an actual JCA PICO scoping/consolidation process [27].

Our review of the identified publications also revealed that although a transparent PICO scheme is included in the HTA reports of some markets (e.g., Germany), the majority of national HTA reports do not routinely include this type of detail. Consequently, insights gathered from a retrospective extraction of the PICOs from HTA reports may differ from learnings gained through a proactive scoping process. There is also uncertainty about how the PICO consolidation will be applied in a real-world JCA scoping process, including whether certain parameters will be used (e.g., minimum number of countries requiring a PICO) to define the eventual list of PICOs. We recognise that the number of PICOs can vary significantly depending on the type of indication; highly dynamic indications, as expected, result in a much higher number of PICOs than indications for sparsely populated, rare diseases, largely driven by comparators and sub-populations in the former category [28]. This was evident in the PICO scoping simulation in a complex indication, which raises critical questions about whether all sub-populations should be included or whether in practice, the number would be refined through consolidation discussions, based on specific criteria/thresholds. This lack of clarity complicates early planning and JCA dossier preparation process from an HTD perspective, when preparatory work to support the submission of a robust JCA dossier needs to begin well in advance of the anticipated dossier submission deadline.

## 5. Conclusions

The limited number of published multi-country PICO scoping exercises, along with their attributes, such as the small number of participating countries, constrains robust analysis and makes it difficult to draw clear conclusions or predict PICO scoping and consolidation outcomes in real-world JCA processes. In particular, there is insufficient data to identify trends, best practices, or common pitfalls in the JCA PICO consolidation process.

Clearer guidance is needed to ensure that HTDs can optimally prepare for submissions, enabling us to better anticipate the relevant PICOs and prepare appropriate evidence ahead of the final assessment scope being shared. HTDs ideally want to ensure that the final set of PICOs included in a JCA assessment scope are both comprehensive and feasible to support robust evidence synthesis and meaningful comparative analysis while minimising the process burden on both assessors and HTDs [29]. The JCA scoping process, including the consolidation phase, should be viewed not merely as a technical exercise but as a strategic dialogue that balances scientific rigour with practical factors such as data availability, clinical relevance, and the diversity of healthcare systems across the EU.

To help achieve this, we propose the following recommendations:

### 5.1. Establishment of Specific Criteria to Guide Identification of Relevant PICOs for JCA

We recommend that a set of criteria are established to guide the selection of appropriate PICOs during the JCA scoping process, based on the following parameters:

- Relevance to Clinical Practice: Comparators identified during the PICO scoping process should reflect treatments routinely used in real-world practice, rather than being determined solely by their inclusion in guidelines. This approach ensures that the scope aligns with actual standard-of-care practices across Member States and is critical for ensuring that JCAs address evidence questions that are meaningful to support national decision-making.
- Validity of distinct PICOs for sub-populations: The inclusion of distinct sub-populations in the PICO process should be based on their clinical relevance and impact on treatment outcomes. Clear guidance is needed on whether a minimum number of countries must request a specific PICO for it to be considered valid.

### 5.2. Provide Greater Clarity on the JCA PICO Consolidation Process

There is a need for greater clarity on how PICOs will be consolidated for JCAs, to help HTDs suitably prepare relevant and proportionate evidence to address the JCA assessment scope. We recommend that clearer guidance is issued outlining the steps in the scoping and consolidation process, including criteria such as number of Member States requesting a specific PICO and how that influences its inclusion, to enable more predictable and efficient dossier preparation.

The current challenges identified based on available JCA PICO scoping and consolidation examples highlight the need for clearer, more inclusive dialogue on this topic. The current lack of clarity surrounding the JCA PICO scoping process risks undermining the EU HTAR’s ambition to deliver harmonised, timely, and high-quality JCAs across Member States. Without clear, consistent guidance on how to define and consolidate PICOs, especially in complex therapeutic areas, there is a risk of misalignment between EU-level assessments and national HTA expectations. Such uncertainties could contribute to delays in evidence generation and slower patient access to innovative treatments, which, if not addressed would undermine the EU HTAR’s goal of improving efficiency, transparency, and equity in health technology evaluation across Europe. By implementing the recommendations outlined above, stakeholders should benefit from increased transparency on the JCA PICO scoping process, ultimately leading to enhanced dossier preparation by HTDs and JCAs that optimally evaluate new technologies in the context of current clinical practice.

## 6. Limitations

The identification and review of published multi-country PICO scoping processes examples was not a systematic literature review, implying the possibility that some relevant publications may have been overlooked.

The PICO scoping simulation based on a NSCLC indication was conducted using a single example and does not precisely replicate previous exercises in terms of population/line of treatment. Therefore, direct comparison of findings is not possible. Additionally, the example chosen was quite complex and may not be representative of other cases, which could lead to a simpler list of PICOs in different contexts.

In practice, the complexity of a JCA depends not only on the predictability and number of PICOs but also on the feasibility and methodologic requirements of analyses, including indirect treatment comparisons (ITCs). A subsequent step in the PICO scoping simulation process would involve evaluating the feasibility of analyses based on the specified PICOs, including estimating the number of analyses and determining where ITCs may be required.

## Figures and Tables

**Table 1:** Details of identified and included publications.

Publication Author, Date	Product: Indications	Number of Included Countries	Total Number of PICOs Identified
Young and Staatz, 2023 [11]	Alosifel: Perianal fistulas in CD	3	3
	Yescarta: DLBCL & PMCL	3	5
	Kymriah: B-Cell ALL & DLBCL	3	B-Cell ALL: 7 DLBCL: 6
	Luxturna: Retinal dystrophy, biallelic RPE65 mutations	3	3
	Zolgensma: SMA; Presymptomatic SMA	3	SMA: 6 pre-symptomatic SMA: 8
	Tecartus: ALL	3	8
	Upstaza: AASC deficiency	3	1

Buchholz et al., 2023 [12]	Osimertinib: NSCLC	5	3
	Pembrolizumab: NSCLC	5	2
	Venetoclax: ALL	5	1

van Engen et al., 2024 [13]	NSCLC	7	10 (14 when UK included)
	Multiple Myeloma	10	16 (18 when UK included)

EUnetHTA-21-PICO Exercise 1 [16]	Prostate Cancer	8	6

Hollard et al., 2024 [14]	Olaparib: Ovarian Cancer	5	4
	Trastuzumab-Deruxtecan: HER2+ BC	5	8
	Venetoclax: CLL	5	6
	Daratumumab: MM	5	4
	Atezolizumab NSCLC	5	8

Jindal and Saharia, 2024 [15]	Epcoritamab: DLBCL	5	6 (UK: 20)
	Loncastuximab tesirine: DLBCL	5	4 (UK: 25)
	Tisagenlecleucel: DLBCL	5	20 (UK: N/A)
	Glofitamab: DLBCL	5	5 (UK: 35)
	Axicabtagene ciloleucel: DLBCL	5	25 (UK: 24)

EUnetHTA-21-PICO Exercise 2 [17]	Epstein–Barr Virus Positive Post-Transplant Lymphoproliferative Disease (EBV + PTLD)	10	5 (1 in the full population and 4 in sub-populations)

EUnetHTA-21-PICO Exercise 3 [18]	Late-Onset Pompe Disease	9	9

EFPIA Report [19]	Product X in metastatic solid tumours	4	7 PICOs
	Product Y in late line hematologic tumours	4	6 PICOs
	Product Z in 1L hematologic tumours	4	23 PICOs

Prada et al., 2024 [20]	Kimmtrak: human leukocyte antigen (HLA)-A*02:01-positive adult patients with unresectable or metastatic uveal melanoma	5	N/A (Consolidation not conducted)
	Fetcroja: Bacterial Infection	5	

Conesa et al., 2024 [21]	polatuzumab-vedotin-DLBCL	4	2

EU HTA CG 1 [22]	Durvalumab (HCC)	22	13

EU HTA CG 3 [23]	Adagrasib (NSCLC)	21	13

EU HTA CG 5 [24]	Etranacogene dezaparvovec (Haemophilia B)	23	6

**Table 2:** Overview of the number of PICOs from the JCA PICO scoping simulation in NSCLC.

	Number of Population(s)	Number of PICOs
Full population (as per aspirational label/per study)	1	7
Sub-populations ^1^	15	60

^1^ sub-populations based on histology (squamous and non-squamous) and specific PD-L1 biomarker cut-off values.

## Data Availability

The original contributions presented in this study are included in the article. Further inquiries can be directed to the corresponding author(s).

## Abbreviations

The following abbreviations are used in this manuscript:

AASC	Aromatic L-Amino Acid Decarboxylase
ALK	Anaplastic Lymphoma Kinase
ALL	Acute Lymphoblastic Leukaemia
ATMPs	Advanced Therapy Medicinal Products
BC	Breast Cancer
CD	Crohn’s Disease
CHMPs	Commission for Human Medicinal Products
CLL	Chronic lymphocytic leukaemia
DLBCL	Diffuse Large B-cell Lymphoma
EBV	Epstein–Barr Virus
EFPIA	European Federation of Pharmaceutical Industries and Associations
EGFR	Epidermal Growth Factor Receptor
EMA	European Medicines Agency
EU	European Union
EU HTAR	European Union’s Regulation on Health Technology Assessment
EUnetHTA	European Network for Health Technology Assessment
HemTums	Hematologic tumours
HCC	Hepatocellular carcinoma
HTA	Health Technology Assessment
HTA CG	HTA Coordination Group
JCA	Joint Clinical Assessment
LoQ	List of Questions
MA	Marketing Authorisation
MM	Multiple Myeloma
MS	Member States
NCE	New Chemical Entity
OPD	Late-Onset Pompe Disease
OvC	Ovarian Cancer
PC	Prostate Cancer
PICO	Population, Intervention, Comparator, Outcome
ROS	Reactive oxygen species
HTD	Health Technology Developer
mNSCLC	Metastatic Non-Small Cell Lung Cancer
NSCLC	Non-Small Cell Lung Cancer
RD	Retinal dystrophy
SMA	Spinal muscular atrophy
SolTum	Solid Tumour
